# High protein and cholesterol intakes associated with emergence of glucose intolerance in a low-risk Canadian Inuit population

**DOI:** 10.1017/S1368980015003080

**Published:** 2015-10-23

**Authors:** Saghar Sefidbakht, Louise Johnson-Down, T Kue Young, Grace M Egeland

**Affiliations:** 1 School of Dietetics and Human Nutrition and Centre for Indigenous Peoples’ Nutrition and Environment (CINE), McGill University, Montreal, Canada; 2 School of Public Health, University of Alberta, Edmonton, Canada; 3 Department of Global Public Health and Primary Care, Faculty of Medicine and Dentistry, University of Bergen, and Norwegian Institute of Public Health, Kalfarveien 31, 5018 Bergen, Norway

**Keywords:** Glucose tolerance, Diabetes, Protein intake, Cholesterol intake

## Abstract

**Objective:**

The rate of type 2 diabetes mellitus among Inuit is 12·2 % in individuals over 50 years of age, similar to the Canadian prevalence. Given marked dietary transitions in the Arctic, we evaluated the dietary and other correlates of not previously diagnosed glucose intolerance, defined as type 2 diabetes mellitus, impaired fasting glucose or impaired glucose tolerance.

**Design:**

Cross-sectional analyses were limited to adults with a completed 2 h oral glucose tolerance test and without pre-existing diabetes. Anthropometric assessments, health and medication usage questionnaires and a 24 h dietary recall were administered.

**Setting:**

Canadian International Polar Year Inuit Health Survey (2007–2008).

**Subjects:**

Inuit adults (*n* 777).

**Results:**

Glucose intolerance was associated with older age and adiposity. Percentage of energy from protein above the Acceptable Macronutrient Distribution Range of 35 %, compared with intake within the range, was associated with increased odds of glucose intolerance (OR=1·98; 95 % CI 1·09, 3·61) in multivariable analyses. Further, cholesterol intake in the highest three quartiles combined (median exposures of 207, 416 and 778 mg/d, respectively) compared with the lowest quartile (median intake of 81 mg/d) was associated with glucose intolerance (OR=2·15; 95 % CI 1·23, 3·78) in multivariable analyses. Past-day traditional food consumption was borderline protective of glucose intolerance (*P=*0·054) and high fibre intake was not significantly protective (*P*=0·08).

**Conclusions:**

The results contribute to the existing literature on high protein and cholesterol intakes as they may relate to diabetes risk.

The prevalence of type 2 diabetes mellitus (DM) was historically rare among Inuit^(^ ^1^ ^)^ but there is evidence that the prevalence is increasing^(^ ^2^ ^,^ ^3^ ^)^, raising concerns that Inuit may face a future epidemic in type 2 DM as experienced by other Indigenous Peoples decades ago^(^ ^4^ ^)^. The traditional diet of Arctic Indigenous Peoples is remarkably high in fat, yet the marine sources of fatty acids are thought to contribute to the historically low prevalence of chronic diseases observed in this population^(^ ^5^ ^)^. Similarly, a study of Canadian Inuit found that while the consumption of traditional food (TF) was high, 66 % of the energy in the diet came from market foods, with the top contributors to energy intake (EI) being refined carbohydrates, bannock (a traditional biscuit), table sugar, cookies and soft drinks^(^ ^6^ ^)^. Inuit TF include marine mammals, game meat, birds, berries and seaweed; foods are traditionally eaten raw.

Lifestyle changes associated with Westernization have increased the prevalence of certain chronic diseases, including obesity, CVD and type 2 DM^(^ ^7^ ^,^ ^8^ ^)^. The nutrition transition in the Arctic coexists with a high prevalence of food insecurity. This represents a dual burden on the population of possible poor diet quality with potential long-term consequences for emergence of diet-related chronic diseases^(^ ^9^ ^)^. Thus, the goal of the current study was to evaluate selected dietary exposures associated with newly ascertained glucose intolerance among Inuit of Canada. Dietary analyses focused upon evaluation of macronutrient intakes and cholesterol, saturated fat, Zn, Mg, fibre and high-sugar beverage consumption given the literature implicating these exposures for development of type 2 DM^(^ ^10^ ^–^ ^18^ ^)^.

## Methods

A cross-sectional Canadian International Polar Year Inuit Health Survey was conducted in three jurisdictions (Inuvialuit Settlement Region of Northwest Territories, Nunavut Territory and Nunatsiavut region in Labrador) in 2007 and 2008^(^ ^2^ ^,^ ^9^ ^)^. Households were randomly selected through randomization of community housing maps and lists and Inuit adults, 18 years of age or older, were invited to participate in the survey. A total of 2796 Inuit households were successfully visited by community research assistants and 1901 (68·0 %) households participated in the survey, with an average of 1·38 participants per household (*n* 2595). Due to survey logistical constraints, approximately 30 % of survey participants had a 75 g, 2 h oral glucose tolerance test (OGTT) either on board the Canadian Coast Guard Ship *CCGS Amundsen* which assisted the research in thirty-three coastal communities or at clinic sites in three land-based surveys for inland communities. Thus, of the original 2595 adults who participated in the survey, only those with a completed OGTT and without pre-existing diagnosed diabetes, as identified by medication or dietary treatments, were included in the present analyses (*n* 777).

The results from the fasting and the OGTT were used to classify participants as glucose intolerant or normoglycaemic, where glucose intolerant included those with impaired fasting glucose or impaired glucose tolerance or diabetes based upon American Diabetes Association criteria^(^ ^19^ ^)^. Fasting (>7·5 h) venous blood samples were collected and kept cool before being centrifuged and frozen at −80°C until later analyses of plasma glucose assessed by the Glucose Hexokinase II method^(^ ^20^ ^)^, handled by Nutrasource Diagnostics (Guelph, Ontario, Canada).

Nurses conducted anthropometric assessments in which participants removed shoes and wore light clothing; weight and percentage body fat (%BF) were measured using a Tanita foot-to-foot bioelectrical impedance scale (TBF-300A; Tanita Corp, Arlington Heights, IL, USA) and height with a portable stadiometer (Road Rod 214 Portable Stadiometer; Seca, Hanover, MD, USA). BMI was calculated as kg/m^2^ where BMI=25·0–29·9 kg/m^2^ was considered overweight and BMI≥30 kg/m^2^ was considered obese according to WHO criteria^(^ ^21^ ^)^. At-risk waist circumference (WC≥102 cm in men, WC≥88 cm in women)^(^ ^22^ ^)^ and at-risk %BF (%BF≥25 for men, %BF≥31 for women)^(^ ^23^ ^)^ were defined using existing guidelines. BMR was calculated by the Tanita scale using fat-free mass (FFM) and the US prediction formula, which was cross-validated in a Japanese population: REE (kcal/d)=20·5×FFM (kg)+462^(^ ^24^ ^)^ (where REE=resting energy expenditure).

Nurses assessed health histories and medication usage, whereas trained bilingual Inuit- and English-language interviewers administered sociodemographic and physical activity questionnaires and a dietary assessment which included a 24 h dietary recall using food portion model kits (Santé Québec, Québec, Canada). A short version of the International Physical Activity Questionnaire was used to calculate each participant’s physical activity score in MET-min/week^(^ ^25^ ^)^ (where MET=metabolic equivalents of task).

Dietary variables based upon the 24 h recalls that were examined included: percentage of energy (%E) from macronutrients, %E from saturated fat, %E from TF, high-sugar drinks (i.e. greater than 25 % of total sugar excluding fruit juices; yes *v*. no) and selected dietary factors postulated to be related to type 2 DM risk, i.e. fibre, cholesterol, Zn and Mg^(^ ^26^ ^)^. The 2007b Canadian Nutrient File was used to estimate the nutrient intakes^(^ ^27^ ^)^. The Canadian Nutrient File does not contain data on glycaemic index of food items and its *trans*-fat updates lag behind the rapid market transitions in product *trans-*fat content. Thus, glycaemic index and *trans*-fat intake were not evaluated in our analyses. TF included local food items that are typically and traditionally harvested in the Arctic including marine and land mammals, fish and seafood, berries and plants including seaweed.

### Statistical analysis

The *χ* ^2^ test was conducted to evaluate differences in demographic characteristics between those with and without glucose intolerance in analyses stratified by sex. Differences in mean dietary exposures between those with and without glucose intolerance were evaluated using the *t* test and results are presented as means with standard deviations. Multivariable logistic regression analyses reporting odds ratios and 95 % confidence intervals were conducted, where the outcome was glucose intolerance (yes *v*. no) and independent variables such as dietary factors, demographic covariates (with tests for sex interactions) and medication usage that could influence glucose intolerance were included. As covariate by sex interaction terms were not significant they were not included in the final models presented. Variables that either were associated with glucose intolerance or changed the beta coefficient of a dietary exposure variable by more than 10 % were retained in multivariable models as covariates. Also, total EI was controlled for in the logistic regressions when evaluating the past-day nutrient intakes such as fibre (g/d), Mg, Zn and cholesterol (mg/d). Macronutrient and TF intakes were evaluated in regression analyses using the nutrient density approach as %E, to control for EI which varies substantially between individuals^(^ ^28^ ^)^. As certain antihypertensive medications can influence glucose values, the use of antihypertensive medications was entered as a covariate in all multivariable analyses^(^ ^29^ ^)^. Regional differences in the degree of acculturation exist in the Arctic; we therefore also adjusted for region (Nunavut, Nunatsiavut and Inuvialuit Settlement Region) in all multivariable analyses.

Further, to evaluate the extent of under-reporting of EI by participants and whether the extent of under-reporting varied by glucose intolerance status, EI:BMR was calculated where a ratio under 1·52 was considered an indication of under-reporting based upon methodology described elsewhere^(^ ^30^ ^)^. Macronutrients were assessed as quartiles of %E and in categories representing above, within and below the Acceptable Macronutrient Distribution Range (AMDR)^(^ ^31^ ^)^. Quartile groups of dietary intake variables were determined and tests for trend were conducted by logistic regression where the median intake level of each quartile group was the independent variable. Because the majority of participants consumed low amounts of fibre and excessive amounts of cholesterol, these dietary variables were consolidated into two categories representing high and low intakes. For fibre, the highest quartile (median intake of 19 g/d) was contrasted against the lowest three quartiles combined (median intakes of 2·6, 6·5 and 10·9 g/d, respectively). For cholesterol, the highest three quartiles (median intakes of 207, 416 and 778 mg/d) were combined into one high cholesterol intake group and contrasted against the lowest quartile (median of 81 mg/d). The diagnostic tests (variance inflation factors and condition indices) for collinearity evaluated the suitability of the final multivariable models presented.

Statistical analyses were conducted using the statistical software package STATA version 11·2. Given that more than one person per household could participate, household was entered as a cluster variable in all multivariable analyses. Two-sided tests were conducted in all analyses and *P*<0·05 was considered statistically significant.

## Results

### Unadjusted analyses of demographic and dietary characteristics

A total of 306 males and 471 females completed the OGTT and the dietary data. The mean age of the men and women included in the present analyses was 42·2 (sd 13·9) years and 41·0 (sd 13·4) years, respectively. The 137 participants with glucose intolerance included six with potential diabetes, eighty-nine with impaired fasting glucose, fifty-two with impaired glucose tolerance, with ten having both impaired fasting and impaired glucose tolerance. The prevalence of glucose intolerance was similar for men (18·6 %) and women (17·0 %) despite significant sex differences in the prevalence of risk factors, where women compared with men were more likely to be obese (44·6 % *v*. 27·7 %; *P*<0·05), have an at-risk WC (63·7 % *v*. 27·8 %; *P*<0·05) and have an at-risk %BF (68·9 % *v*. 40·6 %; *P*<0·05). Conversely, women had lower mean physical activity scores (3180 (sd 3637) MET-min/week) than men (5464 (sd 5105) MET-min/week; *P*<0·05) and smoked fewer cigarettes (10·5 (sd 6·65) per d) than men (12·2 (sd 7·90) per d; *P*<0·05).

In analyses of the percentage of glucose intolerance by categories of risk factors, a striking age gradient in risk for glucose intolerance was noted for men and women (Table 1). Likewise, a greater risk of glucose intolerance was noted among those classified with high BMI, %BF and WC. Those with less than a high school education and those reporting no alcohol drinking had a greater prevalence of glucose intolerance relative to those with a higher level of education and those reporting any alcohol consumption. Alcohol consumption was 8·59 g/d for consumers and 5·68 g/d for the total sample. Smokers had a lower prevalence of glucose intolerance than non-smokers. Family history of diabetes was missing for 25·0 % of men and 22·7 % of women; the percentage of glucose intolerance tended to be higher among those either reporting a family history of diabetes or missing information on family history, with significant group differences noted for men (Table 1).

**Table 1:** Percentage of glucose intolerance by demographic characteristics and sex; Inuit adults (*n* 777), Canada (International Polar Year Inuit Health Survey 2007–2008)

	Men		Women	
Characteristic	*n*	% glucose intolerance	*n*	% glucose intolerance
Region				
Nunavut	212	17·5	314	17·2
Inuvialuit	49	28·6	94	14·9
Nunatsiavut	45	13·3	63	19·1
Age				
<30 years	64	3·1	107	3·7*
30–40 years	72	12·5	105	11·4
>40–60 years	130	22·3	216	20·4
>60 years	40	42·5	43	46·5
Smoking				
Yes	201	14·9*	325	11·7*
No	104	25·0	146	28·8
BMI (kg/m^2^)				
<30·0	226	13·3*	260	10·0*
≥30·0	80	33·8	209	25·4
%BF†				
Low	180	11·1*	145	7·6*
High	123	30·1*	321	20·9*
WC‡				
Low	218	11·5*	169	9·5*
High	88	37·5	303	22·1
Alcohol				
Yes	198	14·7*	276	11·6*
No	92	25·0	174	24·1
Education				
<High school	57	28·1*	74	31·1*
≥High school	246	16·3	396	14·4
Family history of diabetes				
Yes	36	27·8*	90	20·0
No	193	13·5	274	14·6
Missing	77	27·3	107	20·6

%BF, percentage body fat; WC, waist circumference.

* Percentage of glucose intolerance was significantly different by characteristic in analyses conducted separately for men and women (*χ* ^2^ test): *P*<0·05.

† High %BF: %BF ≥31 for women and %BF ≥25 for men.

‡ High WC: WC ≥88 cm for women and WC ≥102 cm for men.

Men reported a total EI of 10 732 (sd 6498) kJ/d and women reported a total EI of 8816 (sd 4376) kJ/d. Values of EI:BMR indicated that participants were mildly under-reporting EI (EI:BMR=1·48 (sd 0·93) and 1·47 (sd 0·77) for men and women, respectively). For men, median total EI was 8887 (interquartile range (IQR) 5046–11 715) kJ/d and 9824 (IQR 6251–14 548) kJ/d for those with and without glucose intolerance, respectively. For women, median total EI was 7590 (IQR 5510–10 652) kJ/d and 8226 (IQR 6046–11 129) kJ/d for those with and without glucose intolerance, respectively.

### Multivariable analyses

In a multivariable logistic regression model entering all statistically significant demographic characteristics, age and WC remained significantly associated with glucose intolerance, whereas smoking, alcohol, family history of type 2 DM and education were no longer associated with glucose intolerance (data not shown). In separate analysis of each macronutrient, adjusting for age, sex, WC, region and use of antihypertensive medications, %E from carbohydrate below the AMDR of 45 % was associated with significant excess risk of glucose intolerance (Table 2). For cholesterol, intake in quartiles 2, 3 and 4 (median intakes of 207, 416 and 778 mg/d, respectively) was associated with a higher risk of glucose intolerance relative to those in the lowest quartile of intake (median 81·0 mg/d; Table 2). Further, a test for trend entering median cholesterol intake for each quartile was significant (*P*=0·00). No other associations or trends were observed for the other nutrients.

**Table 2:** Dietary factors evaluated separately in multiple logistic regression analyses for their association with glucose intolerance; Inuit adults (*n* 777), Canada (International Polar Year Inuit Health Survey 2007–2008)

Variable	Category	Adjusted OR	95 % CI
%E from protein†	AMDR (10–35 %)	Referent	
	<AMDR	0·46	0·17, 1·28
	>AMDR	1·76	0·99, 3·13
%E from fat†	AMDR (20–35 %)	Referent	
	<AMDR	1·22	0·60, 2·49
	>AMDR	0·93	0·61, 1·43
%E from carbohydrate†	AMDR (45–65 %)	Referent	
	<AMDR	1·62*	1·03, 2·56
	>AMDR	1·42	0·62, 3·24
%E from saturated fat†	Recommendation (<10 %)	Referent	
	>Recommendation	1·44	0·97, 2·13
%E from TF†	T1 (median=0)	Referent	
	T2 (median=10·4)	0·90	0·51, 1·58
	T3 (median=38·6)	0·77	0·46, 1·29
Cholesterol (mg/d)‡	Q1 (median=81·0)	Referent	
	Q2 (median=207)	2·10*	1·07, 4·11
	Q3 (median=416)	2·87*	1·46, 5·63
	Q4 (median=778)	3·17*	1·60, 6·27
Fibre (g/d)‡	Q1 (median=2·64)	Referent	
	Q2 (median=6·45)	0·89	0·51, 1·57
	Q3 (median=10·9)	0·93	0·52, 1·67
	Q4 (median=19·0)	0·58	0·28, 1·16
Mg (g/d)‡	Q1 (median=117 )	Referent	
	Q2 (median=190)	1·13	0·62, 2·05
	Q3 (median=268)	1·51	0·80, 2·85
	Q4 (median=426)	1·58	0·69, 3·61
High-sugar drinks†	No	Referent	
	Yes	0·92	0·58, 1·46

%E, percentage of energy; TF, traditional food; AMDR, Acceptable Macronutrient Distribution Range^(^ ^31^ ^)^; T, tertile; Q, quartile.

* Significant OR and 95 % CI: *P*<0·05.

† Model includes each dietary variable separately with age, sex, waist circumference, region and antihypertensive medication usage, with household entered as a cluster variable.

‡ Model includes each dietary variable separately with model 1 variables plus total energy intake.

In further multivariable logistic regression analyses controlling for age, sex, WC, region and use of antihypertensive medications, and simultaneously entering dietary variables, %E from protein above the AMDR *v*. within the AMDR and cholesterol intake in the highest three quartiles combined *v*. the lowest quartile were associated with higher glucose intolerance (Table 3). The result for fibre intake in the highest quartile *v*. the lowest three quartiles combined was not significant (*P*=0·08) and any TF consumption in the past day was marginally significant (*P*=0·054), but both were suggestive of a tendency for a lower odds of glucose intolerance (Table 3, model 1).

**Table 3:** Dietary associates of glucose intolerance in multivariable logistic regression analyses considering demographic and dietary variables simultaneously; Inuit adults (*n* 777), Canada (International Polar Year Inuit Health Survey, 2007–2008)

Variable	Category	Adjusted OR	95 % CI	*P*
Model 1†				
%E from protein	AMDR (10–35 %)	Referent		
	<AMDR	0·57	0·20, 1·61	0·28
	>AMDR	1·98	1·09, 3·61	0·03
Cholesterol (mg/d)	Q1	Referent		
	Q2–Q4	2·15	1·23, 3·78	0·01
Fibre (g/d)	Q1–Q3	Referent		
	Q4	0·61	0·35, 1·06	0·08
%E from TF	T1 (none)	Referent		
	T2–T3 (any)	0·61	0·37, 1·01	0·05
High-sugar drinks	No	Referent		
	Yes	1·43	0·91, 2·26	0·12
Model 2†				
%E from carbohydrate	AMDR (45–65 %)	Referent		
	<AMDR	1·57	0·96, 2·56	0·07
	>AMDR	1·71	0·72, 4·08	0·22
Cholesterol (mg/d)	Q1	Referent		
	Q2–Q4	2·35	1·27, 4·35	0·01
Fibre (g/d)	Q1–Q3	Referent		
	Q4	0·62	0·35, 1·08	0·09
%E from TF	T1 (none)	Referent		
	T2–T3 (any)	0·69	0·43, 1·12	0·13
High-sugar drinks	No	Referent		
	Yes	1·45	0·91, 2·30	0·11

%E, percentage of energy; TF, traditional food; AMDR, Acceptable Macronutrient Distribution Range^(^ ^31^ ^)^; Q, quartile; T, tertile.

† Multivariable models include all dietary variables listed under model 1 and model 2 and age, sex, waist circumference, antihypertensive medication usage and region, with household entered as a cluster variable.

In analyses where we substituted %E from protein with %E from carbohydrate, we found that %E from carbohydrate above *v*. below the AMDR was not associated with glucose intolerance (Table 3, model 2). As %E from protein and %E from carbohydrate were highly and inversely correlated, the variables were not considered together in any multivariable analyses.

All multivariable analyses presented in Tables 2 and 3 were evaluated with additional adjustment for family history of diabetes, smoking, education and alcohol consumption. These additional analyses identified no changes (by more than 10 %) to the beta coefficients presented in Tables 2 and 3. Model 1 was also run with %E from fat but this variable did not contribute significantly to the model and the covariates did not change by more than 10 % as compared with the other models in Table 3.

## Discussion

The dietary findings agree with the literature on dietary risk factors for type 2 DM and they add to our understanding of the nutritional transition that may be important in a population undergoing rapid acculturation.

Dietary characteristics emerged as significant determinants of glucose intolerance in the current cross-sectional health survey. Our findings suggest that high protein and low carbohydrate intakes were associated with greater risk for glucose intolerance. Further, we found a significant test for trend for cholesterol intake in our study population, similar to the Iowa Women’s Health Study in which the highest quintile of cholesterol intake (median of 382 mg/d) was associated with increased risk of incident diabetes in postmenopausal women^(^ ^26^ ^)^. In contrast to the Iowa Women’s Health Study, the intake of dietary cholesterol was excessively high in the current Inuit study population. While cholesterol intake is a risk factor for CVD, its importance in the aetiology of diabetes is not yet established and additional research in this area is warranted.

Our findings are similar to those of the high-risk Sandy Lake Cree of Ontario, Canada in which high protein and low carbohydrate intakes were significantly related to newly identified type 2 DM^(^ ^14^ ^)^. Currently, the existing literature on protein intake and glucose metabolism is inconsistent: a high protein intake, in the short term, may improve glucose metabolism^(^ ^32^ ^)^, but in the Health Professionals Follow-Up Study, high animal protein and fat intakes were associated with an increased risk of diabetes, findings which were attributed to red and processed meat^(^ ^10^ ^)^. In the Women’s Health Initiative, higher protein and energy intakes were associated with the development of diabetes^(^ ^33^ ^)^. In the European Prospective Investigation into Cancer and Nutrition, risk of type 2 DM was higher with higher protein intakes, especially animal protein^(^ ^34^ ^)^. The Alpha-Tocopherol, Beta-Carotene Cancer Prevention Study reported high processed meat consumption was a risk factor for type 2 DM^(^ ^35^ ^)^. Haem Fe intake was positively related to type 2 DM in the Nurses’ Health Study, in the Iowa Women’s Health Study and in Mediterranean and Chinese cohorts^(^ ^36^ ^–^ ^39^ ^)^. However, TF such as marine mammals and game are rich in haem Fe and therefore not a likely candidate for explaining the associations observed in our study population, as we identified an almost significant beneficial association between past-day TF consumption and glucose intolerance.

Also, while more work is needed to identify mechanisms, the emerging literature linking dietary exposures to gut microbiota is a promising area of research. In rats, the amount of total protein rather than the source has been identified as an important determinant of the degree of protein fermentation in the gut, with implications for both the presence of toxic fermentation by-products and the bacterial composition in the intestine^(^ ^40^ ^)^. Also noteworthy is that in a systematic review of the literature red and processed meat has been related to type 2 DM in a number of large cohort studies^(^ ^41^ ^)^. The top contributors to market protein in our study population were red and processed meats, which contain nitrates and nitrites that are absent in TF.

Inuit TF differs from market food in that it provides beneficial nutrient exposures, being a rich source of long-chain *n*-3 fatty acids and antioxidants^(^ ^42^ ^)^; an evaluation of which goes beyond the scope of the current paper. Also, the type of amino acids present in TF and market food protein sources may be important given the observation that cod protein improved insulin sensitivity in insulin-resistant men^(^ ^43^ ^)^. The finding that carbohydrate below the AMDR was associated with greater risk of glucose intolerance cannot be disentangled from %E from protein, given the strong inverse correlation between the two macronutrients (*r*=−0·65). The mechanisms by which cholesterol may play a role in glucose intolerance are not known^(^ ^26^ ^)^, but could be partly attributed to the correlation between animal protein and cholesterol (*r*=0·345). However, when we entered cholesterol in the same model with %E from protein, the effect of cholesterol remained stable and significant and diagnostic tests indicated no collinearity problems.

The current finding that %E from TF was associated with a near significant lower risk for glucose intolerance is compatible with reports from Alaska and Greenland which investigated the association between fatty acid status and type 2 DM^(^ ^5^ ^,^ ^44^ ^–^ ^46^ ^)^ but contradicts another from Greenland that found higher glucose intolerance with TF intake^(^ ^47^ ^)^. Another report from Greenland found that intakes of fruits and seal meat were negatively associated with the risk of diabetes^(^ ^3^ ^)^.

### Limitations

The study, however, is not without its limitations. The study population showed a mild degree of under-reporting of EI overall. Another limitation is that in all observational studies extremes in one dietary exposure variable can be related to extremes in other dietary exposures. Thus, in our observational study, one must interpret the data with caution as the overall findings speak to the potential deleterious aspect of unbalanced diets and cannot provide definitive evidence related to any specific nutrient exposure.

Another limitation of the dietary assessment is the reliance upon one 24 h recall in a relatively small study population. This may explain why the fibre intake in our population, which was very low for the majority of participants, did not reach statistical significance in its association with glucose intolerance. The time-consuming and comprehensive nature of the health survey precluded the use of repeated 24 h recalls, 24 h urinary nitrogen or a comprehensive and detailed FFQ given survey logistics and efforts to minimize research burden for study participants. Finally, the cross-sectional study design precludes the ability to rule out the possibility of reverse causation.

## Conclusion

Nutrition transition continues throughout the Arctic and coincides with many cultural and built infrastructure changes that influence psychosocial stress and lifestyle behaviours which have consequences for an epidemiological transition of increased obesity and chronic diseases. The current study, based upon the rank ordering of study participants based upon one 24 h dietary recall, suggests that high cholesterol and protein intakes are associated with glucose intolerance among Inuit.

Further research is needed to elucidate beneficial and deleterious mediators within diets and their dose–response effects. Knowledge translation activities and other measures to combat obesity and to improve dietary quality are needed to prevent the emergence of cardiometabolic diseases in the Canadian Arctic.
